# Can fish species co-occurrence patterns be predicted by their trait dissimilarities?

**DOI:** 10.1098/rsos.230160

**Published:** 2023-11-08

**Authors:** Ruben D. Cordero, Donald A. Jackson

**Affiliations:** Ecology and Evolutionary Biology, University of Toronto Faculty of Arts & Science¸ Toronto, Ontario Canada, M5S 3G3

**Keywords:** trait-based analysis, null models, aquatic ecology, co-occurrence patterns, lake fish

## Abstract

Trait-based analyses have been successful in determining and predicting species association outcomes in diverse communities. Most studies have limited the scope of this approach to the biotic responses of a small number of species or geographical regions. We focused on determining whether three biologically relevant traits (body size, temperature preference and trophic level) influence the patterns of co-occurrence between multiple species. We used fish species presence/absence from 9204 lakes in Ontario, Canada, to obtain effect sizes of 2001 species-pair co-occurrence values, using a null model approach. Euclidean distances between each species-pair were calculated for each of the three traits selected. Multiple regression models and randomization tests were used to determine the direction and significance of the relationship of each trait with the observed co-occurrence values. The results show that species temperature preference was significantly related to co-occurrence patterns, indicating the effect of environmental filtering. Trophic level was significantly related to co-occurrence values for both linear and quadratic terms, suggesting that segregation between species is driven by large differences in this trait (predation effects). Unexpectedly, body size was not significantly related to the observed co-occurrence patterns. We provide a new approach to test relationships between species assemblages and trait conditions.

## 1. Introduction

A fundamental goal in the field of ecology is to understand which of many diverse factors and mechanisms affect the structure of communities and their internal dynamics, and how these factors contribute to these interspecific and species-environment interactions. Studies have focused on different components of the community structure, such as patterns of species assemblage and abundance [1], turnover [2], nestedness [3], co-occurrence [4,5], food webs [6] and species traits [7]. Additional studies have examined whether those patterns arise from deterministic or neutral processes [8]. Among the aforementioned approaches, the analyses of species co-occurrence have been a subject of extensive interest and debate for the past 40 years, in part because earlier studies described the patterns observed in many natural communities as being the product of biotic interactions among species (typically interspecific competition) [9,10], without appropriately testing the hypothesis [11]. Subsequently, many species co-occurrence analyses incorporated null models, which allowed the testing of the hypothesis that the observed patterns of species distribution are structured non-randomly [11–13].

Different approaches have been incorporated into null models to control for or test the effect of important factors on species co-occurrence patterns. For example, environmental filtering may contribute to which species potentially co-occur due to common habitat conditions and/or geography [14,15], or the effect of the habitat size on co-occurrence patterns [16,17]. More recently, trait-based analyses have been considered to test whether, and to what extent, communities are structured by environmental filtering and/or biotic interactions [18,19]. However, whether species traits are significant predictors of species co-occurrence patterns remains little explored in communities with multiple trophic levels and biotic interactions, with some studies focused on communities of birds [20], invertebrates [21] and plants [22].

**Figure 1.**
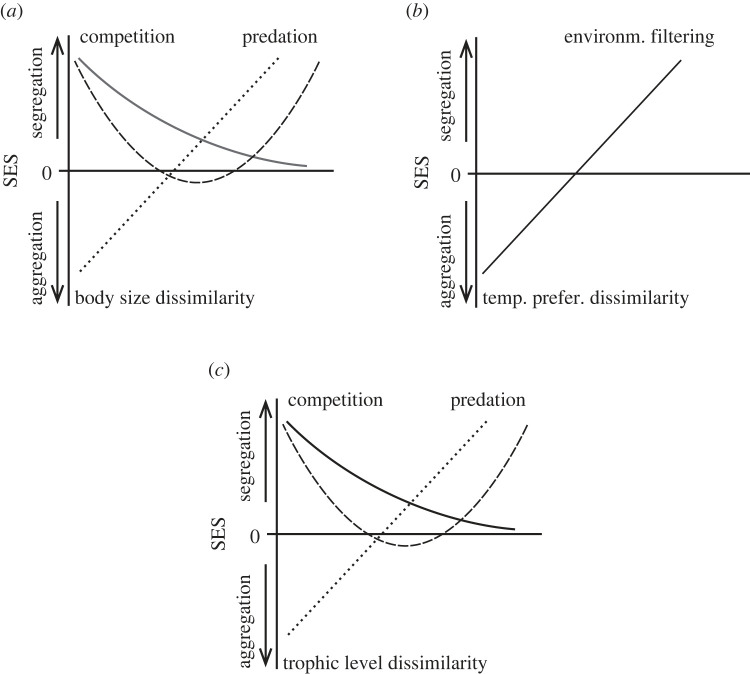
Hypothesized relationships between species-pair co-occurrence patterns and the analysed traits. (*a*) Strong interspecific competition between species with similar body size (left side, solid line) will contribute towards negative co-occurrences (segregation), and this effect decreases when differences in body size increase. Strong predation effects between species with larger differences in body size result in negative co-occurrence (right side, dotted line). The dashed line shows a potential quadratic relationship that occurs when small differences in body size show strong negative co-occurrence (competition signal) and decreases when differences in body size increase, but for species with larger body size differences the segregation increases because of predation effects (right side). (*b*) For temperature preference, aggregation patterns are expected when differences between species for this trait are small due to similar habitat requirements and environmental filtering repeatedly selecting subsets of species (left side). When differences in temperature preference increase, it is expected that segregation between species increases because of environmental filtering (right side). (*c*) For trophic level, it is expected that species with small differences co-occur negatively due to greater effects of competition (left side, solid line). When species trophic levels become dissimilar it is expected that species segregate because of strong predation effects (right side, dotted line). The dashed line shows a quadratic relationship hypothesized to occur when strong segregation patterns between species with similar trophic level (competition) are reduced by the increasing differences that would lead again to high segregation patterns expected because of predation between species with large differences in trophic level.

Trait-based analyses have been used to determine the patterns and mechanisms that structure communities by assessing whether the observed assemblage is consistent with the results expected due to differences in species traits [7,23]. Thus, the type and magnitude of the species co-occurrence patterns may depend on the degree of similarity of their traits [24]. For example, body size has been long recognized as an important factor influencing species interactions, particularly in aquatic ecosystems, as community structure can be affected by the size range of prey that a predator is capable of consuming [25–28]. For lake fish communities, differences in body size may have strong and different effects on the pattern of association observed between species (figure 1*a*). For example, two species with similar body size may co-occur more frequently than expected at random or be aggregated (figure 1*a*, dotted line, left-lower side), due to similar environmental requirements and/or habitat filtering, as observed among minnow species in lakes in Ontario [13,17]. Alternatively, the same species may exhibit a pattern of negative co-occurrence or segregation (figure 1*a*, solid line, left-upper side) when strong interspecific competition for resources occurs. Furthermore, for species with marked differences in body size (e.g. minnows and larger predatory species), a significantly segregated pattern (figure 1*a*, right side, either dashed or dotted lines) may be expected due to strong predation effects [5]. Additionally, a quadratic relationship could be observed if competition is strong between species of similar body size resulting in patterns of segregation (figure 1*a*, dashed line), and this competition-related segregation decreases with increasing differences for this trait, yet interspecific predation can result in segregation when differences in body size become sufficiently large (right side figure 1*a*).

Another important species trait is thermal preference (e.g. cold- versus warm-water fish assemblages, low-alpine versus high-alpine plant assemblages) which has been shown to contribute to structuring the community by selecting or restricting species with different thermal requirements [29–31]. For instance, some variation in fish community composition, between lakes that do not stratify thermally (i.e. shallow lakes) and lakes that do stratify thermally, can be explained through differences in thermal guilds of the species present [32,33]. Thus, when the difference in temperature preference between species is small, abiotic filtering through shared habitat requirements will contribute towards a pattern of aggregation (figure 1*b*, left side) [5]. Conversely, when differences in temperature preference are large, it is expected that those species pairs will have fewer numbers of positive co-occurrence, because of environmental filtering (figure 1*b*, right side).

Likewise, trophic level is a measure representing a key trait that has been used to define guilds, with guilds being defined as a group of species that exploit the same resource [28,34,35]. Trophic level can be useful to identify species pairs that are potential competitors (species within the same guild are presumed to compete more strongly for similar resources) or potentially have a predator–prey relationship (e.g. large piscivorous species and small-bodied fish species). Although trophic level can be related to body size, this relationship may vary, as different studies have found relationships between these traits, ranging from strongly positive through to random relationships [36]. It can be expected that species with similar trophic level (i.e. consuming similar types of food items) may exhibit a negative co-occurrence pattern due to intraspecific competition, or alternatively, show positive co-occurrence, because of similar requirements of food items found in certain habitats if interspecific competition is not high (left side figure 1*c*). By contrast, when species differ greatly in trophic level, a negative co-occurrence pattern may arise, because those species may interact as predator–prey (right side figure 1*c*), which could lead to local prey extirpation when predation effects are very strong [37]. For this trait, it is also possible to observe a quadratic relationship with co-occurrence patterns (figure 1*c*), if the effect of competition is strong between species having similar trophic levels to generate segregation (left side figure 1*c*), which then decreases as differences in trophic levels increase, but segregation increases again when those trait differences become large enough due to a strong predation effect (right side figure 1*c*).

In this study, we examine whether species traits are significantly associated with the observed co-occurrence patterns of lake fishes in Ontario. We determine whether three biologically significant traits (body size, thermal preference and trophic level) can predict the co-occurrence patterns obtained for each species pair. Specifically, we tested the following hypotheses and predictions. 1. The overall relationship of species co-occurrence patterns is non-randomly associated with this combined set of traits. Then considering each trait separately, 2. for body size, it is expected there will be a negative and significant relationship between body-size dissimilarity and co-occurrence patterns if the signal of competition is strong (i.e. species having similar body size tend to compete more strongly and segregate each other), or alternatively a positive and significant relationship if the signal of predation is strong (i.e. species with very different body size tend to have a predator–prey relationship and be segregated). A quadratic relationship will represent a transition from competition to predation effects as body-size differences increase. Random co-occurrence patterns are expected if the effects of competition and/or predation are relatively weak. 3. For temperature preference, we expect a positive and significant relationship with the species co-occurrence patterns if the signal of environmental filtering is strong (i.e. species with more dissimilar temperature preference tend to be more segregated; figure 1*b*). 4. For trophic level, we expect a negative association in the species co-occurrence patterns if competition is strong, or a positive association with co-occurrence patterns if the predation effect is strong. A quadratic relationship will represent a transition from competition to predation effects as trophic-level differences increase. Random association between trophic level and co-occurrence patterns is expected if the effects of competition and/or predation are not strong (figure 1*c*).

## 2. Methods

### 2.1. Location

The province of Ontario, Canada, has more than 250 000 lakes, distributed in an area exceeding one million km^2^, ranging from Mixed Hardwoods Forest in the south and middle region to Subarctic plains in the north [38]. The formation of these lakes started during the Wisconsin Laurentide ice sheet retreat, *ca* 15000 y.a. [39,40]. Administratively within the Province of Ontario these lakes are subdivided into 3 primary, 28 secondary and 144 tertiary watersheds [41]. The aforementioned characteristics make of this region a *natural experiment* in which several studies have elucidated, for instance, post-glacial recolonizing routes [39], geographical patterns of community composition [5,42,43], and the effect of climate change on communities and species ranges [44].

### 2.2. Data collection

**Figure 2.**
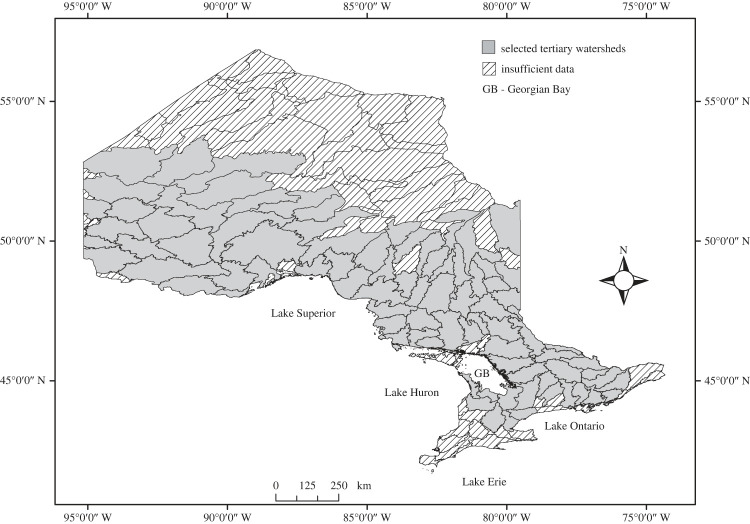
Map of Ontario showing the tertiary watersheds analysed. Selected watersheds contain a minimum of 20 fish species and 20 lakes.

To obtain the species co-occurrence values (i.e. response variable), we used the aquatic habitat inventory (AHI) database, which was a program of the Ontario Ministry of Natural Resources and Forestry. This database includes incidence data (presence/absence) of approximately 100 lake fish species, from almost 10 000 Ontario lakes [41], where each lake was sampled during a single period during a given year (i.e. point-in-time data), and data for individual lakes represent species captured during that sampling period [42,45]. From the AHI database, we removed those watersheds with fewer than 20 lakes and 20 fish species (following Cordero & Jackson [5]), to ensure a matrix with a minimum size of 20 × 20 elements to be randomized [46]. For this study, we used data from 70 fish species, distributed in 9204 lakes, across 86 watersheds (figure 2; electronic supplementary material, table S1), with the watershed used as the unit at which initial analysis was done (i.e. each watershed represents an incidence matrix).

The predictor variables of trait data for adult fish species were obtained from multiple databases, and include body size, temperature preference and trophic level. For body size, we obtained data of total length from the Ontario Freshwater Fishes Life History Database [47]. For temperature preference, we used the values calculated by Hasnain *et al*. [48] for Ontario lake fish, and missing values for some species (see electronic supplementary material, table S1) were estimated by averaging temperature values from congeneric species. For trophic level, we used the values gathered in the FishBase [49]. These databases are regularly updated based on new studies and the data used in this study were obtained during 2021. Some trait values in the aforementioned databases were updated based on more detailed and recent reports (see electronic supplementary material, table S1).

### 2.3. Data analysis

Co-occurrence values (response variable) were obtained following Cordero & Jackson [5]. We used the species presence–absence (i.e. 1/0) matrix within each watershed to calculate the observed C-score values, which measures the level of negative co-occurrence or segregation between two species [12]. The C-scores were obtained for all the species pairs individually in each watershed (unit of analysis). Then, each incidence matrix (i.e. watershed) was evaluated by generating a distribution of C-scores (i.e. 999 sets of randomizations) under the null model of fixed row and column totals, using the curveball algorithm [46] included in the EcoSimR package [50]. We obtained the standard effect sizes (SES of the C-score), using the formula:

$$\mathrm{SES}=\frac{\left(C_{\mathrm{obs}}\text{–}C_{\exp}\right)}{{\mathrm{SD}}_{\exp}},$$

where *C*_obs_ represents the *C*-score of each species pair, *C*_exp_ represents the mean value of the distribution of randomly generated *C*-scores and SD_exp_ is the standard deviation of the randomly generated C-scores. Thus, because the C-score measures relative segregation between species, positive SES values indicate negative co-occurrence or segregation between species (i.e. species tend to co-occur less than expected by chance) and negative SES values indicate positive co-occurrence or aggregation between species (i.e. species tend to co-occur more than expected by chance). Finally, we calculated the mean SES for each species pair across the watersheds where they occur, which represents the overall co-occurrence value of such species pair in our study system.

To obtain the species-pair trait-distance values (predictor variables), we standardized trait values (converted to *z*-scores) to ensure a comparable effect across the traits in the models, and then calculated the Euclidean distance between species for each trait (i.e. a separate distance matrix for each trait). The Euclidean distance between species for each trait was calculated using the vegan package in R [51]. We generated a dataset that includes species co-occurrence values (SES) as the response variable plus three predictor variable columns with between-species distances in body size, temperature preference and trophic level. A multiple regression model was used to determine whether and to what degree co-occurrence values (SESs) can be explained by trait-distance values. This approach summarizes the overall relationship between the predictor and the response variables, and to determine the relative importance of each predictor variable explaining the response variable, while controlling for the effects of the other predictors [52,53]. To test the statistical significance of the underlying relationships, we performed a randomization test, where we held constant the combination of the three trait distance values per species pair, while randomizing, 9999 times, the co-occurrence values (SES) of the species pairs [54,55]. Randomizations were done in a manner that preserved the underlying trait distance relationships between species and is effectively a randomization of the underlying species identities, as in the Mantel test [56]. The next steps tested the four proposed hypotheses:

1. To test the hypothesis that overall species co-occurrence patterns are non-randomly related to the set of selected traits, we included the SES of the co-occurrence values of all 2001 species pairs and all trait categories in the multiple regression. Both linear and quadratic forms of the terms for body size and trophic level are included to test the competing hypotheses. The slope coefficients estimated by the multiple regression model, and the *p*-value generated by the randomization test were used to assess whether the relationship between each trait and the co-occurrence values differed from random.
2. To test the second hypothesis that negative co-occurrence patterns are associated with either similar (competition signal) or different (predation signal) body size, we used a multiple regression model to detect significance between the co-occurrence values and the body size distances of all species pairs. We included both the linear term and the quadratic term of body size as variables, to test the potential linear or quadratic relationships between this trait and co-occurrence patterns (figure 1*a*).
3. To test the third hypothesis (negative co-occurrence patterns because of environmental filtering and positive patterns because of shared habitat requirements), we used a regression model to test for a significant relationship between the co-occurrence values and the temperature preference distances of all species pairs (i.e. 2001 pairs).
4. To test the fourth hypothesis that negative co-occurrence patterns are associated with either similar (competition signal) or different (predation signal) trophic level, we used a multiple regression model to test for significance between the co-occurrence values and the trophic level distances of all species pairs (i.e. 2001 pairs). We included both the linear and the quadratic term of trophic level as variables, to test the potential linear and quadratic relationships of this trait with co-occurrence patterns (figure 1*c*).

We report the probability values associated with the formal tests but consider the estimates from the randomization test to be a more appropriate significance assessor. The data represent distances between species and therefore distance values may not be independent of one another.

## 3. Results

We analysed 70 species and their trait values (body size, temperature preference and trophic level; electronic supplementary material, table S1), from which we estimated the relationships between the co-occurrence values (SES) and trait-distance values for 2001 species pairs. The results of the multiple regression model for hypothesis 1 showed a negative slope for body size (slope = −0.04), although the randomization test did not show the slope differed from random (randomization-based *p* = 0.52) (table 1). The quadratic term of body size (slope = −0.04, *p* = 0.01) was non-significant in the randomization test (randomization-based *p* = 0.24), discounting a quadratic relationship between this trait and the co-occurrence values (table 1). There is no evidence supporting a meaningful general relationship between body size and co-occurrence patterns across all analysed species pairs. For temperature preference, the multiple regression resulted in a positive significant slope (slope = 0.13, randomization-based *p* = 0.005), indicating that species pairs with greater differences in temperature preference (e.g. those from different temperature guilds) tend to co-occur less than expected by chance (species segregation) (table 1). For the trophic level trait, the multiple regression model results produced a negative and non-significant slope (slope = −0.06, randomization-based *p* = 0.44 for this trait) (table 1). However, the quadratic term of trophic level exhibited a positive and significant coefficient (slope = 0.1, randomization-based *p* = 0.027), which indicates higher levels of segregation when species have lower or higher differences in trophic level and less segregation for species with medium differences of this trait (figure 1 dashed line). The results for hypothesis 1 indicate that temperature preference and trophic level have a significant association on their observed co-occurrence patterns of all species pairs, but for body size this effect did not differ from random expectations.

**Table 1.** Results of the multiple regression model and the randomization test for all species pairs (2001 species pairs, hypothesis 1). The model included all the variables (terms). Italic font values show significant traits in the randomization test. The quadratic form of terms is indicated by (QT).

	estimate slope	standard error	*t*-value	*p*-value regression model	*p*-value randomization test
body size	0.1	0.05	1.31	0.2	0.52
body size (QT)	−0.04	0.014	−2.7	0.01	0.24
temperature preference	0.13	0.023	5.61	<0.0001	*0.005*
trophic level	−0.06	0.06	−0.94	0.35	0.44
trophic level (QT)	0.1	0.02	3	0.003	*0.027*

**Figure 3.**
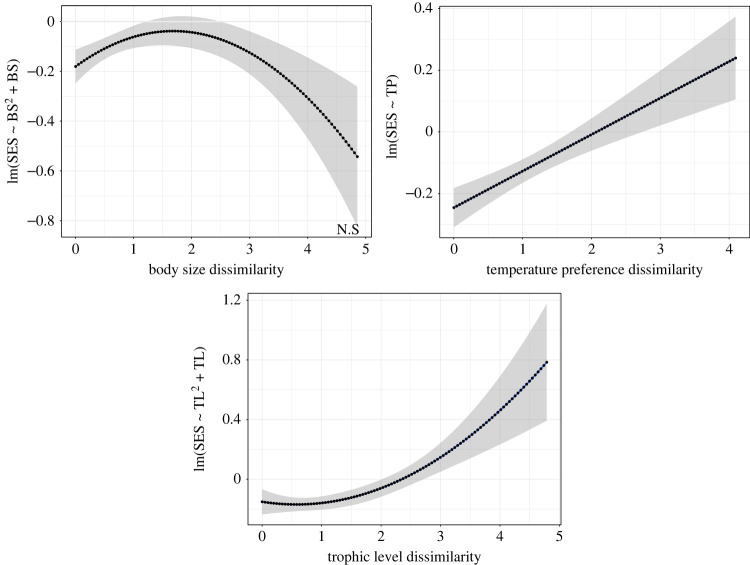
Results of regression model trend lines for individual traits observed between the selected traits and the co-occurrence values. The horizontal axis shows the standardized distance values between species for each trait, and the vertical axis shows the co-occurrence values calculated individually for each model. (*a*) The regression line between body size and co-occurrence values shows a negative quadratic trend, but non-significant relationship (N.S) detected by the permutation test. (*b*) A positive and significant linear trend is observed between temperature preference distance and co-occurrence values. (*c*) A positive and significant relationship between both linear and quadratic terms of trophic level and co-occurrence values. Note that axes are not at the same scale for the different trait plots.

For hypothesis 2 (effect of body size on co-occurrence patterns), the slope of the regression model indicates a positive relationship between body size distances and co-occurrence patterns, although this relationship was non-significant using the randomization test (slope = 0.16, randomization *p* = 0.14; table 2). In addition, the quadratic term of body size exhibits a negative slope (figure 3*a*) that was non-significant according to the regression model (slope = −0.05, randomization *p* = 0.13; table 2).

**Table 2.** Results of the single regression model (individual trait analysis) and the permutation test for all species pairs (2001 species pairs, hypotheses 2, 3 and 4). Italic font values show significant traits in the permutation test. Quadratic terms (QT) were tested within the same analysis with the distance values of body size and trophic level.

	estimate slope	s.e.	*t*-value	*p*-value regression model	*p*-value permutation test
body size	0.16	0.05	3.02	0.003	0.14
body size (QT)	−0.04952	0.01421	−3.484	0.001	0.13
temperature preference	0.12	0.023	5.145	2.96×10^–7^	*0.009*
trophic level	0.12	0.023	5.34	1.02×10^–7^	*0.006*
trophic level (QT)	0.04	0.01	5.7	1.4×10^–8^	*0.01*

For hypothesis 3 (effect of temperature preference on co-occurrence patterns), we obtained a positive and significant trend (figure 3*b*) in co-occurrence values with increasing differences in temperature preferences (slope = 0.12, randomization-based *p* = 0.009; table 2).

For hypothesis 4 (effect of trophic level on co-occurrence patterns), the regression model showed a positive and significant slope (slope = 0.12, randomization-based *p* = 0.006; table 2) between distances of this trait and the co-occurrence values across all species pairs. Moreover, the test for the quadratic term of trophic level was also significantly related to the co-occurrence values across all species pairs (randomization-based *p* = 0.01; table 2). The combination of the linear and quadratic terms shows a shift from aggregation for species pairs with small differences in trophic level through to increasingly segregated co-occurrence patterns for species pairs having large differences in trophic level (figure 3*c*).

## 4. Discussion

Understanding the influence of ecologically relevant traits on species associations is key in determining the structure of natural communities [20,57]. In this study, we assessed the influence of three biologically relevant species traits (body size, temperature preference and trophic level) on the observed co-occurrence patterns of multiple fish species pairs across thousands of lakes in Ontario, Canada. In the overall model including all species-pairs data, we found species co-occurrence values were significantly related to only the linear term relationship for temperature preference and both linear and quadratic terms for trophic level differences, but for body-size, both the linear and its quadratic term were not statistically significant. Temperature preference can represent environmental filtering imposed on some species in particular habitats (lakes), while trophic level can indicate biotic effects some species impose on others (e.g. predators on prey). Contrary to our expectations, body size trait values were not significantly associated with the species co-occurrence patterns. Temperature preference exhibits a significant positive slope, indicating that species with larger differences of this trait tend to exhibit more segregation. Interestingly, the results for trophic level support both linear and quadratic relationships between pairwise distances of this trait and co-occurrence patterns. These results suggest that species co-occurrence patterns could be predicted based on the difference (i.e. distance) in some biologically relevant species traits, which provides more detailed information about the structure of natural communities.

Body size is a key trait structuring and predicting metacommunities [58,59]. Moreover, body size is correlated with several other species traits that affect the structure and dynamics of ecological networks, across multiple levels of organization, which have been valuable to assess changes in local and regional biodiversity [60]. For example, global geographical co-occurrence patterns observed in mammals were significantly related to body size, along with phylogenetic relationships, demonstrating the importance of considering this trait in analyses of geographical patterns of species distribution and biodiversity [61]. However, the results of our study, contrary to our expectation under hypothesis 2, indicate that body-size differences are not significantly related to the fish species co-occurrence values across the body size categories observed in Ontario lakes. A possible explanation is that body size in Ontario fishes is weakly related to food-web position and not all the larger species are piscivorous [62]. For example, most adult catostomids (suckers and redhorses) and Common Carp (*Cyprinus carpio*) have large bodies but are not piscivorous. Nine out of the 16 fish species having larger body size (greater than 70 cm) were classified as secondary consumers (not strictly piscivorous), which may explain the aggregated or random co-occurrence patterns with smaller fish species (contrary to expected) that could be considered as prey items for larger species, solely due to body size differences. This inconsistency between body size and trophic position could be the product of morphological constraints, like gape size, although gape size is commonly related to body size. Some large-bodied fish species, like the Common Carp, have a small gape size and sub-terminal mouth that limits the variety of items they are capable of ingesting [63]. Thus, positive or random co-occurrence patterns observed between species with large differences in body size could dilute the strong negative co-occurrence values observed in other species pairs involved in strong predator–prey interactions (*sensu* Cordero & Jackson [5]). When habitat (lake) size is large enough to provide environmental heterogeneity, adequate refuge for small-bodied species, and suitable environmental conditions for larger species (e.g. Northern Pike *Esox lucius*, Lake Trout *Salvelinus namaycush*, bass *Micropterus* *spp*.), a wide range of species may co-occur resulting in a breakdown of the strong predator–prey signal found in smaller lakes [5,17].

The temperature at which a species performs physiologically best is known as its temperature optimum or preference [48], and this measure can be used as proxy to determine the effect of environmental filtering in structuring communities [64,65]. In our study, differences in temperature preference were significant and positively related to the co-occurrence values when analysing all fish species pairs in Ontario lakes and there was an increase in median SES values observed along the analysed temperature preference categories. This outcome is consistent with environmental filtering as species having similar temperature requirements show higher levels of co-occurrence or aggregation than expected and species pairs with large differences in temperature preference show increased patterns of segregation. For Ontario fish species, it has been observed that temperatures above the preferendum can have a stronger filtering effect, limiting the range of species that are not adapted to warm-water conditions (and correlated lower dissolved oxygen concentrations) [26,66,67].

Temperature conditions have important implications in limiting the geographical range of species (including potentially invasive species) from the range of more susceptible communities that may be impacted by the arrival of such species. For instance, Smallmouth Bass (*Micropterus dolomieu*) is a top predator that has a northern boundary in Ontario delimited by the 16°C isotherm of the July mean air temperature [40,68]. This fish species is known to have strong negative effects on many small fish species via predation [5,69], and even large-bodied species like Lake Trout, by reducing their food availability [70,71]. Thus, under various climate-change scenarios where temperatures are projected to increase over this century [72,73], especially in northern latitudes, it may be expected that Smallmouth Bass continues to expand its northern range, reaching new northern lakes and impacting the local fish communities by extirpating native species in those habitats [40,44,65]. Therefore, the approach presented in this study may provide a means to identify and assess the potential impact on biodiversity and abundances of the communities of such changing range distributions, due to the significant influence of temperature preference on co-occurrence patterns of Ontario Lake fishes.

Trophic level has been considered, in general, to be positively correlated with body size [28,36] and similar results were expected for both traits in our study. The significant results obtained for both the linear and quadratic terms of trophic level, support a more complex relationship between trophic level and co-occurrence patterns (figure 1*c*, dashed line). There is a generally weak pattern of aggregation for small differences in trophic level, but this changes towards segregated co-occurrences as differences in trophic level increase. Interspecific competition has been often identified as a major factor structuring communities by segregating species that are outcompeted by stronger species [10,74,75]. For example, an experimental foraging study between two competitor fish species, Bluegill (*Lepomis macrochirus*) and Golden Shiner (*Notemigonus crysoleucas*), found that Bluegill outperformed Golden Shiner [76]. However, in natural habitats other factors may influence the magnitude of competition, like differences in species population sizes [77], habitat size [17] or species foraging preferences (e.g. generalists versus specialists) [75]. As well, predation has been long identified as a key factor structuring communities [5,37,78,79] and the magnitude of this factor is expected to increase with differences in trophic level. For example, Cordero & Jackson [5] found consistent high-segregation levels between top fish predators (i.e. Northern Pike, Smallmouth Bass, Largemouth Bass) and minnows (e.g. Northern Redbelly Dace *Chrosomus eos*, Fathead Minnow *Pimephales promelas*).

Unlike body size, trophic level is less affected by additional factors, such as gape size or mouth orientation, that affect the type of consumed items (e.g. detritus, invertebrates, fish) expected for large-bodied species [80,81] and therefore may represent a more direct assessment of diet. However, unlike the increasing segregation pattern observed when differences in temperature preference become larger across the analysed categories, the pattern observed for trophic level was not consistently increasing across the trait differences. At low to intermediate differences in trophic level, the co-occurrence relationships are effectively weak aggregation or random, but become strongly segregated at larger trophic-level differences. This trend is consistent with what we predicted under the hypothesis of predation-induced patterns of segregation, like what was found by Cordero & Jackson [5], for a subset of the taxa examined in the current study. Therefore, trophic level differences appear to support the predation-based hypothesis, rather than a competition-based one, in determining patterns of species co-occurrence.

## 5. Conclusion

In summary, this study presents an approach to assess the association between different biologically relevant traits and the co-occurrence patterns of multiple fish species pairs from thousands of lakes across a large geographical scale. We found results consistent with the hypothesis that temperature preference exerts an effect on co-occurrence patterns related to environmental filtering mechanisms, where species with larger differences in this trait tend to co-occur negatively (segregated). The effect of trophic level on co-occurrence patterns was significant and the combination of both linear and quadratic relationships observed suggest that segregation patterns are consistent with the outcome due to strong predation effects. However, contrary to what we expected, body size difference was not significantly related with the co-occurrence patterns, perhaps due to several large-bodies species having lower trophic level positions due to benthic feeding traits, rather than piscivory. The approach developed here provides a framework to incorporate more biologically relevant traits in future co-occurrence analysis to test other traits that may influence community patterns of co-occurrence. As well, the choice of traits must be tailored for the specific communities of study, e.g. appropriate traits for plant communities or zooplankton communities will clearly be different from each other.

## Data Availability

The original dataset of presence/absence belongs to the Aquatic Habitat Inventory (AHI), which is a program from the Ontario Ministry of Natural Resources and Forestry and can be requested directly from its office. We have permission to use these data but not to share them. We present the resulting data and specifics regarding data choices (e.g. omitted lakes and/or species) in the electronic supplementary material [82].
